# Antimicrobial consumption, costs and resistance patterns: a two year prospective study in a Romanian intensive care unit

**DOI:** 10.1186/s12879-017-2440-7

**Published:** 2017-05-22

**Authors:** Carmen Axente, Monica Licker, Roxana Moldovan, Elena Hogea, Delia Muntean, Florin Horhat, Ovidiu Bedreag, Dorel Sandesc, Marius Papurica, Dorina Dugaesescu, Mirela Voicu, Luminita Baditoiu

**Affiliations:** 1 Microbiology Department, “Victor Babeş” University of Medicine and Pharmacy, 16 Victor Babeş, Timişoara, Romania; 2 Pius Branzeu” Emergency Clinical County Hospital, Timişoara, Romania; 3 Regional Center of Public Health Timisoara, Timişoara, Romania; 4 Anesthesiology and Intensive Care Department, “Victor Babeş” University of Medicine and Pharmacy, Timişoara, Romania; 5 Pharmacology and Clinical Pharmacy Department, “Victor Babeş” University of Medicine and Pharmacy, Timişoara, Romania; 6 Epidemiology Department, “Victor Babeş” University of Medicine and Pharmacy, Timişoara, Romania

**Keywords:** ICU, Antibiotic, DDD, ESBL, MRSA

## Abstract

**Background:**

Due to the vulnerable nature of its patients, the wide use of invasive devices and broad-spectrum antimicrobials used, the intensive care unit (ICU) is often called the epicentre of infections. In the present study, we quantified the burden of hospital acquired pathology in a Romanian university hospital ICU, represented by antimicrobial agents consumption, costs and local resistance patterns, in order to identify multimodal interventional strategies.

**Methods:**

Between 1^st^ January 2012 and 31^st^ December 2013, a prospective study was conducted in the largest ICU of Western Romania. The study group was divided into four sub-samples: patients who only received prophylactic antibiotherapy, those with community-acquired infections, patients who developed hospital acquired infections and patients with community acquired infections complicated by hospital-acquired infections. The statistical analysis was performed using the EpiInfo version 3.5.4 and SPSS version 20.

**Results:**

A total of 1596 subjects were enrolled in the study and the recorded consumption of antimicrobial agents was 1172.40 DDD/ 1000 patient-days.

The presence of hospital acquired infections doubled the length of stay (6.70 days for patients with community-acquired infections versus 16.06/14.08 days for those with hospital-acquired infections), the number of antimicrobial treatment days (5.47 in sub-sample II versus 11.18/12.13 in sub-samples III/IV) and they increased by 4 times compared to uninfected patients. The perioperative prophylactic antibiotic treatment had an average length duration of 2.78 while the empirical antimicrobial therapy was 3.96 days in sample II and 4.75/4.85 days for the patients with hospital-acquired infections. The incidence density of resistant strains was 8.27/1000 patient-days for methicilin resistant *Staphylococcus aureus*, 7.88 for extended spectrum β-lactamase producing *Klebsiella pneumoniae* and 4.68/1000 patient-days for multidrug resistant *Acinetobacter baumannii.*

**Conclusions:**

Some of the most important circumstances collectively contributing to increasing the consumption of antimicrobials and high incidence densities of multidrug-resistant bacteria in the studied ICU, are represented by prolonged chemoprophylaxis and empirical treatment and also by not applying the definitive antimicrobial therapy, especially in patients with favourable evolution under empirical antibiotic treatment. The present data should represent convincing evidence for policy changes in the antibiotic therapy.

## Background

The intensive care unit (ICU) is often called the epicentre of infections, due to its extremely vulnerable patients, the wide use of invasive devices and broad-spectrum antimicrobials, which favours the emergence of multidrug resistance (MDR) [1–3]. The prognosis of patients who develop hospital-acquired infections (HAI) in the ICU is poor and the mortality rates are higher if it involves an MDR organisms [4]. Inappropriate use of broad-spectrum antimicrobials is frequent, partly because of unwarranted prescriptions of antimicrobials, which may be caused by uncertainty regarding the type of infection, among other possible explanations [5].

The prevalence of MDR bacteria, especially Gram negative bacilli, such as, extended spectrum β-lactamase (ESBL) producers, has increased, not just in Europe, but also in other areas of the world [4, 6, 7]. In South-Eastern Europe the percentage of MDR *Klebsiella pneumoniae* was 25–50%. As well as that, *Pseudomonas aeruginosa* strains resistant to 3 or more antibiotic classes had their highest level of incidence levels in Romania, Bulgaria and Greece (with percentages being between 25 and 50%) and the incidence of MDR *Acinetobacter* spp. reached a peak in Italy, Greece and Portugal, with slightly lower percentages (under 50%) in Bulgaria, Romania, and Hungary [8].

In Romania, according to the European Antimicrobial Resistance Surveillance Network data, in 2012, the most frequently reported hospital-acquired (HA) isolates were: *Staphylococcus aureus* (19%), with 54.5% of these being methicillin-resistant (MRSA), *K. pneumoniae* (13.5%) with 42.27% MDR strains and *Acinetobacter baumannii* (12%) of which 86.27% were resistant to carbapenems [9].

ICUs have expenses estimated to reach as much as 20% of a hospital’s budget and therefore they represent the largest clinical costs for hospitals. The European Community reported a cost of 1.5 billion Euros and 25,000 deaths related to infections caused by MDR bacteria [10, 11].

The aim of the present study was to quantify the burden of HA pathology in a Romanian university ICU, and to assess the actual ICU consumption and financial costs of antimicrobial agents relative to the patients’ pathology, and went on to identify the local resistance patterns, in order to identify multimodal interventional strategies in this unit.

## Methods

### Study design

Between the 1st of January 2012 and the 31st of December 2013, a prospective study was conducted in the largest ICU in western Romania i.e. a department with 27 beds dedicated to both surgical and nonsurgical pathologies in a public regional hospital with a total of 1100 beds.

### Data collection

According to Romanian law, all hospitals are obliged to collect data continuously on HAI and antimicrobial resistance and to report these findings as part of passive or sentinel surveillance systems to local public health authorities and further to the National Institute of Public Health.

Data collection for the present study was based upon the electronic database of the Microbiology Laboratory and of the Pharmacy Department and also on the data taken from the patients’ observation charts every 2 days. The approval of the Ethics Committee at “Pius Branzeu” Timisoara Emergency Clinical County Hospital was requested and granted: no.44346/11.12.2012.

### Sampling

All patients admitted to the ICU for the study period and who received antibiotic treatment were included and were monitored from admission until either discharge, transfer, or death. Patients with an ICU stay of under one hour were excluded, as were those under 18 years of age. Consecutive readmissions were considered in the case of discharged patients who were later readmitted on different occasion.

Four sub-samples of patients were considered, according to the basis for their antibiotic treatment:

- SI – patients with non-infectious diseases who only received prophylactic antibiotherapy (peri-surgical treatment). None of these patients had a presumptive diagnosis of infection upon admission, nor did they require microbiologic diagnosis tests;
- SII – patients with community acquired infections (CAI) or with infectious complications of chronic diseases which were clinically manifest at the time of ICU admission, and for which they received antibiotics;
- SIII – patients who developed HAI 48 h or more after ICU admission, as well as patients with HA pathology with onset in other hospital departments and who, due to their evolution, required transfer to the ICU;
- SIV – all cases of CAI at the moment of admission, complicated by HAI;

### Variables

HAI were defined according to national legislation i.e. Order of the Ministry of Health no. 916/2006 and according to Centre of Disease Control and Prevention (CDC) definitions [12]. In HA pneumonia (that occurs 48 h or more after admission, which was not incubating at the time of admission) we also included ventilator-associated pneumonia (that arises more than 48–72 h after endotracheal intubation). Blood stream infections (BSI) included sepsis established by laboratory tests, clinical sepsis, HA-BSI from infected central catheter and secondary sepsis following other primary infection sites. The category of HA urinary tract infections also included sub-clinical infections – cases of bacteriuria in the presence of urinary catheters, without clinical symptoms but for which antimicrobial treatment was administered. Surgical site infections from SIII and SIV include superficial and deep postoperative infections.

The consumption of antimicrobial drugs included antibacterial substances (J01 code of the Anatomical Therapeutic Chemical - ATC), tuberculosis specific drugs (J04) and anti-fungal drugs (J02), excluding antiviral medication (J05). Anti-fungals were included because examination revealed a frequent association between antibacterial and anti-fungal treatments in SIII and SIV.

Consumption was carefully monitored for the period of ICU stay, in Defined Daily Dose (DDD)/1000 patients-days, according to the method established by the WHO Collaborating Centre for Statistical Pharmacologic Methodologies (WHO ATC/DDD Index 2015). DDD is an internationally acknowledged unit of measure representing the average daily dose of antimicrobial administered to an adult weighing 70 kg [13].

To identify the cost/patient-day we used the prices provided by the National Catalogue of prices during the studied period for medicines approved for human use and authorised for the Romanian market. Costs were further converted into €, at an exchange rate of 4.44 RON for 1€ (the arithmetic mean of the medium yearly exchange rate in 2012 and 2013, established by the National Bank of Romania).

### Microbiological methods

Bacterial identification and antimicrobial sensitivity tests were performed using the Vitek 2 automated system (bio-Mérieux, Marcy-L’Etoile, France). Susceptibility category was identified according to the CLSI breakpoints. *P. aeruginosa* ATCC 27853, *Escherichia coli* ATCC 25923, *S. aureus* ATCC 25922 reference strains were used as controls in the antimicrobial sensitivity tests.

A clone strain was defined as a strain of the same bacterial species, with the same antibiotic susceptibility pattern, isolated in the same patient during one month, regardless of the biological product in which it was isolated and it was excluded to avoid duplication. MDR was defined as acquired non-susceptibility to at least one agent in three or more antimicrobial categories.

The percentage of resistant strains was calculated by dividing the number of resistant strains by the total number of strains of the same species (which were tested for that specific antibiotic) multiplied by 100. The incidence density of resistant strains was defined as the number of resistant strains per 1000 patient-days.

### Statistical analysis

Continuous numeric variables were described by the mean value, and the nominal ones by frequency counts and percentage. Numerical data distribution was tested for normality with the Kolmogorov-Smirnov test. Comparison of dichotomous variables was performed by the chi-square test with Fischer correction. Numeric variables with normal distribution were compared with the t test for independent samples and those with non-Gaussian distribution were compared using the nonparametric Mann-Whitney test. Multiple comparisons were planned in advance during the design phase and no additional testing or adjustments were subsequently made.

Statistical significance was calculated by two-tailed tests and significance threshold was set at *p* values <0.05. The statistical analysis was performed using the EpiInfo version 3.5.4 (Atlanta, USA: CDC) and SPSS version 20 (Armonk, NY: IBM Corp).

## Results

From 1st January 2012 to 31st December 2013, a total of 1596 subjects were included in our study.

The descriptive statistics for the entire study sample is presented in Table 1. The presence of HA pathology doubled the length of stay and the number of antimicrobial treatment days, as seen from the comparative analysis of the four sub-samples, presented in Table 2. In addition, a prolonged period of chemoprophylaxis and empirical antimicrobial therapy should be noted among the infected sub-samples (SII, SIII and SIV).

**Table 1:** Descriptive statistics of the study sample

Characteristic	Value	95% CI
Patients (N1 = 1596)		
Mean age [years]	60.21	59.33–61.08
Male [n (%)]	959 (60.09)	57.6–62.5
Female [n (%)]	637 (39.91)	37.5–42.4
Hospital admissions (N2 = 1696)		
Non-infectious pathology [n (%)] - SI	697 (41.09)	38.7–43.5
Community acquired infections [n (%)] - SII	344 (20.28)	18.4–22.3
Non-infectious pathology complicated by HAI [n (%)] - SIII	609 (35.91)	33.6–38.3
CAI complicated by HAI [n (%)] - SIV	46 (2.71)	2.0–3.6
Average no. days of hospital stay [days]	9.20	8.61–9.79
Deaths [n (%)]	766 (45.16)	42.8–47.6
Improved evolution [n (%)]	857 (50.53)	48.1–52.9
Stationary evolution [n (%)]	52 (3.06)	2.3–4.0
Aggravated evolution [n (%)]	20 (1.18)	0.7–1.9
Transferred cases [n (%)]	1 (0.06)	0.0–0.4
Average no. days of antimicrobial drugs treatment [days]	6.59	6.20–6.99
Number of administered antimicrobial drugs [no.]	1.85	1.78–1.92
Biological samples (N3 = 1291)		
Bronchial aspirate [n (%)]	532 (41.21)	38.5–44.0
Blood [n (%)]	285 (22.07)	19.9–24.5
Urine [n (%)]	167 (12.93)	11.2–14.9
Wound secretion [n (%)]	116 (8.98)	7.5–10.7
Catheter tip [n (%)]	90 (6.97)	5.7–8.5
Peritoneal fluid [n (%)]	32 (2.48)	1.7–3.5
Pus [n (%)]	25 (1.93)	1.3–2.9
Cerebrospinal fluid [n (%)]	14 (1.08)	0.6–1.9
Other [n (%)]	30 (2.32)	1.6–3.3
Isolated strains of species/genera – after excluding duplicates (N4 = 1322)		
*Staphylococcus aureus* [n (%)]	241 (18.23)	16.2–20.5
*Klebsiella pneumoniae* [n (%)]	220 (16.64)	14.7–18.8
*Escherichia coli* [n (%)]	138 (10.43)	8.9–12.3
*Pseudomonas aeruginosa* [n (%)]	137 (10.36)	8.8–12.2
*Proteus mirabilis* [n (%)]	136 (10.28)	8.7–12.1
*Acinetobacter baumannii* [n (%)]	134 (10.13)	8.6–11.9
*Candida albicans* [n (%)]	100 (7.56)	6.2–9.1
Coagulase-negative *Staphylococcus* [n (%)]	85 (6.43)	5.2–7.9
*Providencia stuartii* [n (%)]	23 (1.73)	1.1–2.6
*Proteus spp.* [n (%)]	22 (1.66)	1.1–2.6
*Enterobacter cloacae* [n (%)]	17 (1.28)	0.8–2.1
*Enterococcus faecalis* [n (%)]	12 (0.91)	0.5–1.6
*Serratia marcescens* [n (%)]	12 (0.91)	0.5–1.6
Other [n (%)]	45 (3.40)	2.5–4.6
Resistance phenotypes		
MRSA [n (%)]	129 (53.53)	47.0–60.0
ESBL *Klebsiella pneumoniae* [n (%)]	123 (55.91)	49.1–62.6
MDR *Acinetobacter baumannii* [n (%)]	73 (54.47)	45.7–63.1
ESBL *Proteus mirabilis* [n (%)]	65 (47.79)	39.2–56.5
MRCNS [n (%)]	53 (62.35)	51.2–72.6
MDR *Pseudomonas aeruginosa* [n (%)]	52 (37.96)	29.8–46.6
ESBL *Escherichia coli* [n (%)]	23 (16.67)	10.9–24.0
ESBL *Enterobacter cloacae* [n (%)]	5 (29.41)	10.3–56.0

**Table 2:** Comparative analysis of the sub-samples’ characteristics

Item	SI	SII	SIII	SIV	SI-II p	SI-III p	SI-IV p	SII-III p	SII-IV p	SIII-IV p
Average age [years(95% IC)]^(a)^	58.23 (56.88-59.58)	62.90 (60.96–64.82)	61.32 (60.04–62.60)	60.10 (54.76–65.45)	<0.001	0.004	0.345	0.165	0.325	0.623
Male [n(%)]^(b)^	432 (61.98)	191 (55.52)	368 (60.43)	21 (45.65)	0.045	0.565	0.027	0.139	0.206	0.049
Female [n (%)]^(b)^	265 (38.02)	153 (44.47)	241 (39.57)	25 (54.35)						
Average no. days of ICU stay [days(95% IC)] ^(c)^	4.11 (3.83–4.40)	6.70 (6.05–7.34)	16.06 (14.68–17.43)	14.08 (10.69–17.47)	<0.001	<0.001	<0.001	<0.001	<0.001	0.926
Average no. days of antimicrobial drugs therapy [days(95% IC)] ^(c)^	2.78 (2.62–2.93)	5.47 (4.96–5.96)	11.18 (10.28–12.06)	12.13 (9.31–14.95)	<0.001	<0.001	<0.001	<0.001	<0.001	0.232
Average no. days of empirical therapy [days(95% IC)] ^(c)^	2.78 (2.62–2.93)	3,96 (3.65–4.27)	4.75 (4.48–5.01)	4,85 (3.89–5.81)	<0.001	<0.001	<0.001	0.035	0.132	0.915
Average no. days of definitive therapy [days(95% IC)]	/	6,25 (5.24–7.27)	11.46 (10.24–12.68)	11,53 (8.61–14.46)	/	/	/	0.005	0,001	0.449
Number of a antimicrobial drugs administered in the ICU [no.(95% IC)]^(b)^	1.23 (1.19-1.27)	1.69 (1.58–1.60)	2.57 (2.42–2.72)	2.91 (2.36–3.47)	<0.001	<0.001	<0.001	<0.001	<0.001	0.114
Number of antimicrobial drugs administered in the ICU as empirical therapy [no.(95% IC)] ^(b)^	1.23 (1.19–1.27)	1,24 (1.18–1.29)	1.40 (1.35–1.46)	1,36 (1.21–1.52)	0.754	<0.001	0.026	<0.001	0.055	0.846
Number of antimicrobial drugs administered in the ICU as definitive therapy [no.(95% IC)] ^(b)^	/	1,84 (1.63–2.05)	2.45 (2.27–2.64)	2,93 (2.35–3.52)	/	/	/	0.004	<0.001	0.111
Deaths [n (%)] ^(b)^	196 (28.12)	179 (52.03)	361 (59.27)	30 (65.21)	<0.001	<0.001	<0.001	0.030	0.092	0.428
Improved evolution [n (%)] ^(b)^	475 (68.15)	155 (45.06)	211 (34.64)	16 (34.78)	<0.001	<0.001	<0.001	0.001	0.187	0.985
Stationary & aggravated evolution [n (%)] ^(b)^	26 (3.73)	9 (2.62)	37 (6.08)	0 (0)	0.348	0.048	0.397	0.016	0.606	0.100

^(a)^t-test was applied

^(b)^chi-square test was applied

^(c)^Mann-Whitney test was applied

**Fig. 1:**
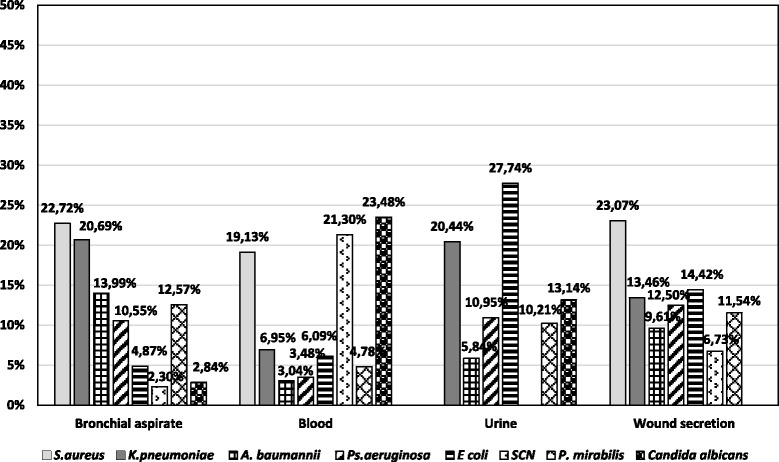
Distribution of the main infection sites

*S. aureus* was isolated mainly from the wound secretions, blood and bronchial aspirates, as shown in Fig. 1. The second most frequently identified was *K. pneumoniae*, isolated mainly from bronchial aspirates, urine and wound secretions, while non-fermentative germs prevailed in bronchial aspirates, wound secretions and urine. We noticed a high percentage of *Candida albicans* in blood cultures, which explains the need to associate anti-fungal preparations in the treatment of BSI.

**Fig. 2:**
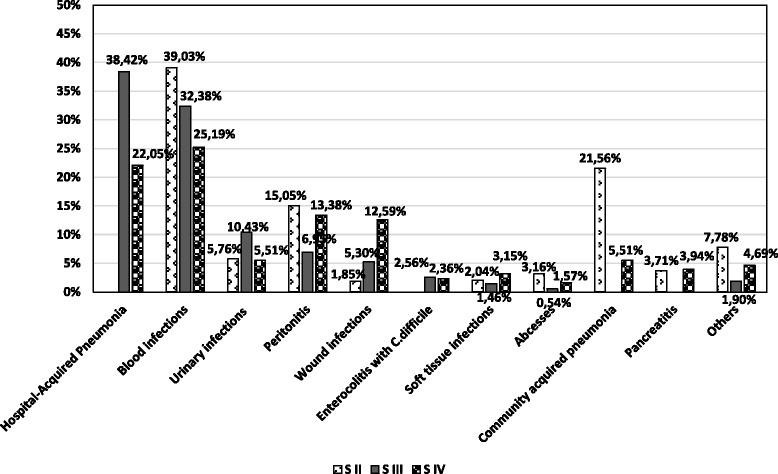
Distribution of the main species isolated according to biological samples

In SII, BSI were predominant (39.03%), followed by community-acquired pneumonia (21.56%), while in the sub-samples with nosocomial pathology (SIII, SIV), HA pneumonia (38.42%/22.05%) and BSI (32.38%/25.19%) prevailed, as shown in Fig. 2. It is to be observed that peritonitis cases came third in SII (15.05%) as well as in SIV (13.38%), as postsurgical infections of the perivisceral areas.

The consumption of antimicrobial agents was 1172.40 DDD/ 1000 patient-days (Table 3), with the following distribution: 13.16% for antibiotic prophylaxis, 15.72% for CAI patients, 64.53% for patients with HAI, and the remaining 6.59% for those with associated community acquired and HA pathology. Antibacterial drug consumption reached the value of 1080.38 DDD/1000 patient-days (with 14.19% in SI, 15.83% in SII, 63.64% in SIII and 6.33% in SIV).

**Table 3:** Consumption on classes of antibacterial & anti-fungal chemotherapeutic agents

Class	DDD / 1000 PATIENT-DAYS				
	Total	S I	S II	S III	S IV
Cephalosporins	264.19	87.75	37.63	133.68	5.13
I-st generation	0	0	0	0	0
II-nd generation	46.96	27.35	4.98	14.50	0.13
III-rd generation	198.19	57.74	31.97	103.54	4.93
IV-th generation	13.43	1.51	0	11.92	0
V-th generation	5.60	1.15	0.67	3.72	0.06
Carbapenems	244.59	8.16	53.84	160.36	22.23
Glycopeptides	132.94	6.13	20.23	96.97	9.61
Polymyxins	115.47	1.15	3.03	103.02	8.27
Penicillins	109.61	20.46	13.47	73.30	2.38
Fluoroquinolones	93.72	13.48	22.42	51.95	5.87
Anti-fungal drugs	92.02	0.91	13.29	68.98	8.84
Imidazole derivatives	27.54	4.67	4.93	16.74	1.20
Aminoglycosides	26.55	4.29	3.22	17.63	1.41
Lincosamides	19.68	6.34	2.75	10.53	0.06
Glycylcyclines	15.67	0	4.68	8.81	2.18
Antimycobacterials	9.74	0	0	0	9.74
Oxazolidinones	8.49	0.64	0.32	7.53	0
Sulfonamides	7.16	0	4.54	2.31	0.31
Macrolides	2.18	0.26	0	1.92	0
Aminophenols	1.52	0	0	1.52	0
Tetracyclines	0.90	0	0	0.90	0
Monobactams	0.43	0	0	0.43	0
TOTAL antibacterial treatments	1080.38	153.33	171.06	687.6	68.39
TOTAL antibacterial & anti-fungal treatments	1172.40	154.24	184.35	756.58	77.23

Regarding the costs of treatments, this reached 9.40 €/patient-day for prophylaxis, 33.14 €/patient-day for CAI, 37.20 €/patient-day for HAI and 46.93 €/patient-day for those with associated community acquired and HA pathology.

As for the financial cost, 73.11% of the total value of antibacterial and anti-fungal treatment was allocated to patients with HA pathology to which an additional 6.11% was added for the patients in SIV. Only 5.43% of the amount was represented by antibiotic prophylaxis and 15.35% by the treatment of patients with CAI.

## Discussion

The National Romanian report on HAI for 2012, shows the duration of antibiotic prophylaxis exceeded 24 h in 91.83% of cases, and around half of the patients received antibiotics for prophylactic purposes for more than 3 days [9]. In our study, both the average duration of chemoprophylaxis (2.78 days: minimum 1, maximum 13 days) and empirical therapy (4.75/4.85 days: minimum 1, maximum 20 days) are longer in HA sub-samples (SIII, SIV). This situation is also found in other countries in the region, as can be seen in a Turkish study published in 2013, which identified a perioperative prophylactic antibiotic treatment duration of 4.74 days (minimum 1 and maximum 17 days) [14]. In our case, we noticed situations when clinicians avoid de-escalation, especially in patients with favourable evolution under empirical antibiotic treatment.

Mortality for the entire sample in our study was 45.16% (with variations from 28.12% in SI, to 52.03% in SII, 59.27% in SIII and 65.21% in SIV). A possible explanation for increased mortality in our study could be the high incidence of BSI, even in SII and also the fact that approximately 50% of the patients studied had neurosurgical or neurological pathology (cranio-cerebral trauma, polytrauma, strokes, aneurysms, etc.) with high levels of lethality, evident even in SI, consisting of patients without an infectious disease diagnosis. In the first report from the Care-ICU programme for improved infection control, published in 2008, ICU mortality varied widely between 6% and 48.4%, with a median value of 14.5% [15]. In another study, mortality in an ICU department in Turkey was as high as 63% [16].

In our ICU, BSI occupied the first place (33.90%), followed by respiratory diseases (33.22%), abdominal infections (9.89%) and urinary tract infections (8.70%). The predominance of BSI is also to be found in other studies, like in a Greek study, where the infection distribution was: 36.1% BSI, 25.3% ventilator associated pneumonia, 18.7% surgical infections, 10.4% infections associated with vascular catheter and 9.5% urinary tract infections [17, 18].

The consumption of antimicrobial drugs identified in our study is similar to that mentioned in other studies in the same geographic area. In a project conducted in 130 European hospitals antibiotic consumption went up to 792 ± 147 DDD/1000 patient-days. [19] A lower DDD (87.8/100 bed-days) was reported in a Turkish point prevalence study, published in 2013, on 21 ICU beds, with a cost of 29.95 $ per infected patient [14]. In our study, it is to be noticed the preponderant use of carbapenems in the therapy of infected patients, with the increase in selective pressure, which constitutes a worrying aspect, as the majority of MDR *A. baumannii* also presented carbapenem resistance.

Even though carbapenems should not be used for prophylactic purposes, in our study we found 8.16 DDD per 1000 patient-days used in SI, despite the fact that the patient file did not contain any mentions on clinical symptoms or microbiologic tests to support a diagnosis of infection. Still, it is common ICU practice to administer antibiotics to patients with systemic inflammatory reaction, even in the absence of any evidence of infection.

The data in the literature varies regarding costs. In a Turkish university hospital, the average cost of antibacterial treatment was 89.64$, with higher values for patients with secondary infections, and meropenem was the most expensive of the used drugs (as was the case in our study) [20]. The overall average daily cost of antimicrobial treatment in 310 patients with BSI in a Belgian ICU was 114.25 €, with higher values in patients with HAI and the most expensive were the treatments for BSI with *Candida* spp. [20, 21].

In 2012–2013, as compared with 2005, the incidence density of resistant strains, in the same ICU, doubled for MRSA strains (reaching 8.27/1000 patient-days) and for ESBL *K. pneumoniae* (7.88/1000 patient-days) [15]. A particular aspect in the strains isolated in our study shows the high incidence of *Proteus mirabilis* strains, with many multiple resistant strains (the density of ESBL strains was 4.17/1000 patient-days).

After identifying this situation, the prevention of emergent resistance attributable to selection pressure by a rational antibiotic therapy policy, as well as the prevention of clone spreading by adherence to hospital epidemiology and hygiene principles are mandatory. At the level of our ICU the following measures have been taken:

- antibiotic prophylaxis and empirical therapy protocols which have been previously used in the ICU were revised according to the detected circulation patterns and to the identified consumption of antibacterials;

- GeneXpert®, Instrument Systems was acquired, allowing rapid qualitative in vitro diagnosis by real-time polymerase chain reaction of MRSA infections and colonisation, carbapenem-non-susceptible bacteria, vancomycin resistant enterococci, and even the detection of *Clostridium difficile* infection. This measure has led a more judicious use of antibiotics and massive reducing of empirical therapy period;

- a campaign of hand hygiene intensification for ICU medical staff was set up, using logistical measures (increasing the number of dispensers with antiseptic solutions in each ward), increased training of medical personnel about the importance of hydro-alcoholic rubbing and increasing control of microbial load of the palmar skin;

- 5 steps central venous catheter insertion and maintenance protocols have been introduced, with correct evaluation and confirmation of infection, to avoid antibiotic treatment in the absence of clear evidence of infection or treatment for microbial colonisation in the absence of clinical symptoms;

- a hospital antibiotic committee has been created - currently in Romania, the law requires the compulsory operation of such committees in hospitals, overseeing prescriptions made by infectious disease specialists and if needed, suggesting alternative therapy;

- oversight of antibiotic consumption, by calculating the DDD/100 patient-days hospitalisation was established;

- screening of patients on admission to the ICU has been set up, regarding carriage of MDR bacteria, to reduce the circulation of endemic strains;

- a training course has been organized for ICU specialists, focussing on 24 h postoperative chemoprophylaxis reduction, on avoiding the same antimicrobials in therapy and prophylaxis, the use of long enough T_1/2_ preparations and reduced spectrum, avoiding the use of routine prophylactic use of vancomycin, carbapenems etc.

- training of clinical microbiology staff has also been intensified, highlighting on reporting resistance phenotypes, but training of infection control specialists there has also been improved, enhancing oversight and control of HAI;

One limitation of our study is that it was performed in a single location and may be biased by particular characteristics, given the fact that location may show high epidemiologic differences and a corresponding lack of generalization possibilities. Lower antibiotic doses applied in cases of renal and hepatic failure might alter the total value expressed as DDD/1000 patient-days. Moreover, the antibiotic intake prior to ICU admission, with the potential effect on the incidence of MDR strains was also not assessed.

## Conclusions

The economic burden of antimicrobial treatment of the sub-samples associated with HAI had risen to about 80% the total costs of antimicrobial treatment of the study group. Some of the most important circumstances collectively contributing to increasing the consumption of antimicrobials and high incidence densities of MDR bacteria in our ICU are represented by prolonged chemoprophylaxis and empirical treatment and also by not applying the definitive antimicrobial therapy, especially in patients with favorable evolution under empirical antibiotic treatment. The present data should represent convincing evidence for policy changes in the antibiotic therapy.

## Abbreviations

ATC: Anatomical Therapeutic Chemical
BSI: Blood stream infections
CAI: Community acquired infections
CDC: Center for Disease Control and Prevention
DDD: Defined Daily Dose
ESBL: Extended spectrum β-lactamase
HA: Hospital acquired
HAI: Hospital acquired infections
ICU: Intensive care unit
MDR: Multidrug resistance
MRCNS: Methicillin-resistant coagulase negative staphylococcus
MRSA: Methicillin-resistant *S. aureus*
